# Metastasis to the breast from an adenocarcinoma of the lung with extensive micropapillary component: a case report and review of the literature

**DOI:** 10.1186/1746-1596-5-82

**Published:** 2010-12-17

**Authors:** Nicoletta Maounis, Maria Chorti, Stella Legaki, Eleni Ellina, Aphrodite Emmanouilidou, Maria Demonakou, Xanthi Tsiafaki

**Affiliations:** 1 Department of Clinical Cytology, Sismanoglio General Hospital, Athens, 151 26, Greece; 2 Department of Pathology and Laboratory Medicine, Drexel University College of Medicine, Philadelphia, 19102, PA, USA; 3 Department of Pathology, Sismanoglio General Hospital, Athens, 151 26, Greece; 4 Department of Respiratory Medicine, Sismanoglio General Hospital, Athens, 151 26, Greece

## Abstract

Breast metastasis from extra-mammary malignancy is rare. Based on the literature an incidence of 0.4-1.3% is reported. The primary malignancies most commonly metastasizing to the breast are leukemia-lymphoma, and malignant melanoma. We present a case of metastasis to the breast from a pulmonary adenocarcinoma, with extensive micropapillary component, diagnosed concomitantly with the primary tumor. A 73-year-old female presented with dyspnea and dry cough of 4 weeks duration and a massive pleural effusion was found on a chest radiograph. Additionally, on physical examination a poorly defined mass was noted in the upper outer quadrant of the left breast. The patient underwent bronchoscopy, excisional breast biopsy and medical thoracoscopy. By cytology, histology and immunohistochemistry primary lung adenocarcinoma with metastasis to the breast and parietal pleura was diagnosed. Both the primary and metastatic anatomic sites demonstrated histologically extensive micropapillary component, which is recently recognized as an important prognostic factor. The patient received chemotherapy but passed away within 7 months. Accurate differentiation of metastatic from primary carcinoma is of crucial importance because the treatment and prognosis differ significantly.

## Background

The National Cancer Institute of the U.S.A. estimates, that based on current rates, 12.7% of women born today will be diagnosed with breast cancer in their life time [1]. Although, primary breast cancer is the most common malignancy of adult females, metastatic involvement of the breast is rare with a reported frequency of 0.4 - 1.3% in clinical series [2-5]. Despite its rarity, metastatic disease to the breast is an important diagnostic clinical dilemma, because its treatment differs greatly from that of primary breast cancer.

Sitzentfrey, in 1907, was the first to publish a case of ovarian carcinoma metastatic to the breast [6]. Since then a wide variety of malignancies have been reported to metastasize to the breast and according to the literature the most common primary tumors are melanomas and haematological malignancies [5,7]. Despite the fact that the lung is the most common cancer site in terms of incidence and mortality there are only few published cases on pulmonary carcinomas metastasizing to the breast [8-12].

Carcinomas with micropapillary components have been reported at several anatomical sites, including the breast, urinary bladder, ovary and major salivary glands [13]. The micropapillary component is being increasingly recognized as a prognostic predictor in lung adenocarcinomas and according to many authors it may be a manifestation of aggressive behaviour [14,15]. We report a patient with metastasis to the breast from a pulmonary adenocarcinoma with extensive micropapillary pattern diagnosed concomitantly with the primary tumor.

## Case Presentation

**Figure 1:**
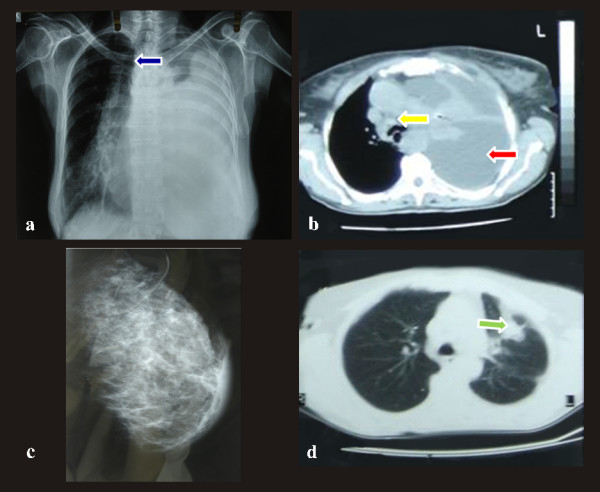
**Imaging techniques**. a) *Chest x-ray*: Massive pleural effusion occupying most of the left hemithorax with evident displacement of the mediastinum to the right (blue arrow). b) *Chest computed tomography: *The left lung is atelectatic and compressed by massive pleural effusion (red arrow). The mediastinum and the trachea are severely displaced to the right. A few lymph nodes can be seen, deeply in the left axilla. Additionally, some paratracheal lymph nodes can be observed (yellow arrow). c) *Chest computed tomography: *A 3,5 × 4,5 cm peripheral lesion, on the left upper lobe, with relative abnormal contour and extension into the surrounding parenchyma. The tumor is in contact to the splanchnic pleura and approached the parietal pleura, possibly invading it (green arrow). d) *Digital mammography: *Diffuse asymmetrical density in the subalveolar region and the upper outer quadrant of the left breast.

A 73-year-old, non-smoker, housewife presented to the emergency department with dyspnea and dry cough of 4 weeks duration. Examination of the chest revealed reduced breath sounds and percussion dullness at the left hemithorax. Physical examination also revealed a painless, poorly defined mass, associated with skin redness, in the upper outer quadrant of the left breast. Palpable left axillary lymph nodes were also noted. A chest radiograph showed massive pleural effusion occupying most of the left hemithorax (Figure 1a). In the chest computed tomography (Figure 1b), the left lung was atelectatic and compressed by massive pleural effusion.

The mediastinum and the trachea were severely displaced to the right. A few lymph nodes deeply in the left axilla and some paratracheal lymph nodes were observed. Clinically the diagnosis was considered to be either a primary breast tumor with lung and pleural metastasis or two synchronous primaries. Mammography showed diffuse asymmetrical density in the subalveolar region and the upper outer quadrant of the left breast. (Figure1c). Additionally, skin thickening was demonstrated in the affected area. Calcifications were not observed. The differential diagnosis included inflammation, lymphoma and inflammatory breast carcinoma. Excisional biopsy was recommended.

Moreover, the patient underwent bronchoscopy which revealed submucosal infiltration causing widening of the secondary carina and obstruction of the orifice of the lingula at approximately 70%. Pleural effusion re-accumulated rapidly so in order to perform pleural drainage and chemical pleurodesis medical thoracoscopy was carried out. During the procedure biopsies were obtained from the parietal pleura. Chest computed tomography (Figure 1d) followed and showed a 3,5 × 4,5 cm peripheral lesion, on the left upper lobe, with relative abnormal contour and extension into the surrounding parenchyma. The tumor was in contact to the splanchnic pleura and approached the parietal pleura, possibly invading it. Finally, an excisional breast biopsy was performed.

Our patient received 4 courses of Bevacizumab, Cisplatin and Docetaxel with no clinical response. Unfortunately the patient died 6 months following diagnosis.

### Cytologic and Immunocytochemical findings

**Figure 2:**
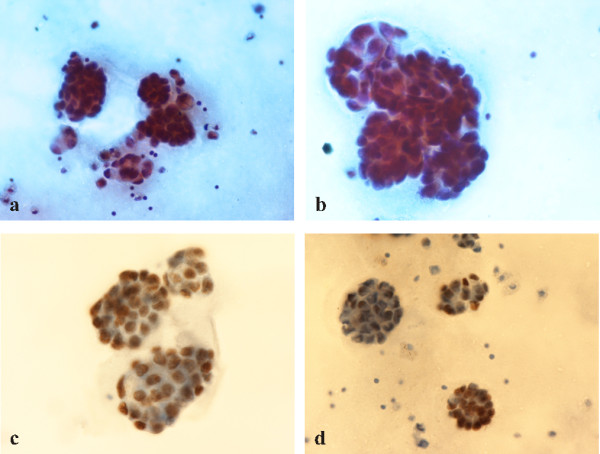
**Pleural effusion aspiration smears**. a) Clusters of malignant cells with morphological features of adenocarcinoma (Papanicolaou stain, ×400). b) Clusters of malignant cells with morphological features of adenocarcinoma (Papanicolaou stain, ×600). c) Immunocytochemical positivity to TTF-1 (×350). d) Immunocytochemical positivity to TTF-1 (×280).

All cytologic specimens were stained by the Papanicolaou technique and evaluated by cytology. By examining the pleural effusion (Figure 2a, b), bronchial washing (BW)s and bronchial brush (BB) specimens the diagnosis of adenocarcinoma was achieved. By immunocytochemistry performed on smears prepared from the pleural effusion sample, tumor cells were strongly immunoreactive for thyroid transcription factor-1 (TTF-1) (Figures 2c, d) and monoclonal CEA. Tumour cells were negative for CK 5/6, estrogen receptors (ER), CA-125 and thyroglobulin (Table 1).

**Table 1:** Antibodies employed in the immunohistochemistry applied

Antigen	Clone	Source, location	Dilution
TTF-1	8G7G3/1	Dako, Glostrup, Denmark	1:50
SP-A	PE-10	Dako, Glostrup, Denmark	1:20
CEA monoclonal	II-7	Dako, Glostrup, Denmark	1:50
ERa	1D5	Dako, Glostrup, Denmark	1:30
GCDFP-15	23A3	NeoMarkers, Fremont, CA, USA	Prediluted
Mammaglobin	304-1A5	Dako, Glostrup, Denmark	1:100
CK 5/6	D5/16B4	Dako, Glostrup, Denmark	1:50
Calretinin	DAK-Calret 1	Dako, Glostrup, Denmark	1:100
CA-125	M11	Dako, Glostrup, Denmark	1:100
Thyroglobulin	DAK-Tg6	Dako, Glostrup, Denmark	1:100
CD 15	MMA	NeoMarkers, CA, USA	Prediluted

TTF-1, thyroid transcription factor-1; SP-A, surfactant apoprotein A; CEA, carcinoembryonic antigen; GCDFP-15, gross cystic disease fluid protein-15; ER, estrogen receptor.

### Histopathological and Immunohistochemical findings

**Figure 3:**
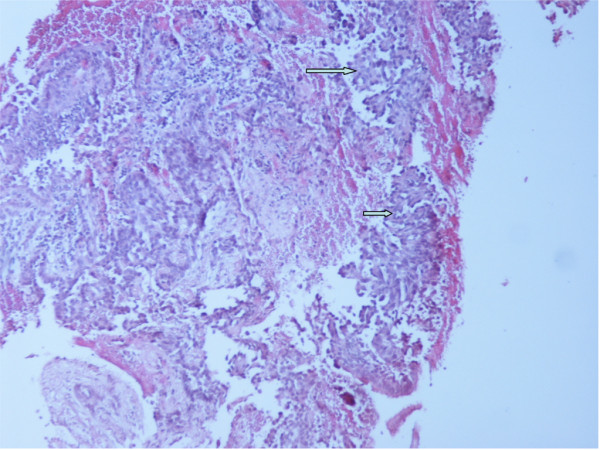
**Bronchoscopy biopsy**. Low differentiated adenocarcinoma with micropapillary component (arrow). (Hematoxylin-eosin, ×100).

Hematoxylin - Eosin (H&E) stained paraffin sections of the bronchoscopy biopsy demonstrated bronchial mucosal infiltration by a low differentiated adenocarcinoma. An extensive micropapillary component was identified (Figure 3). The latter was observed as papillary structures with tufts that lacked a central fibrovascular core. Additionally, occasional psammoma bodies were noted. Our differential diagnosis included primary lung adenocarcinoma, metastatic adenocarcinoma from the thyroid, the breast or the ovary and finally metastatic - epithelioid (papillary) type - mesothelioma. The tumor cells demonstrated immunoreactivity for CD 15 (Leu-M1), TTF-1, Surfactant protein A (SP-A) and monoclonal CEA. The neoplastic cells lacked expression of gross cystic disease fluid protein-15 (GCDFP-15), ER, mammaglobin, CK 5/6, calretinin, CA-125 and thyroglobulin (Table 1). Based on the histology and the immunohistochemical staining pattern, a diagnosis of primary lung adenocarcinoma with micropapillary component was rendered.

**Figure 4:**
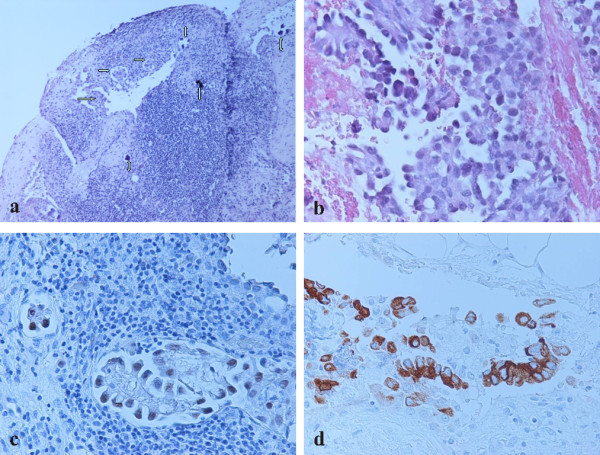
**Parietal pleural biopsy**. a) Infiltration by adenocarcinoma with micropapillary pattern (single arrow). Multiple psammoma bodies are observed (double arrow). (Hematoxylin-eosin, ×100). b) Infiltration by adenocarcinoma with micropapillary pattern. (Hematoxylin-eosin, ×400). c) Immunohistochemical nuclear TTF-1 positivity of malignant cells. (×400). d) Immunohistochemical cytoplasmic SP-A positivity of malignant cells. (×400).

H&E stained paraffin sections of the parietal pleura biopsies revealed diffuse infiltration by malignant epithelioid type cells, which demonstrated solid and micropapillary pattern. Additionally, numerous psammoma bodies were observed (Figures 4a, b). Tumor cells revealed the same immunoprofile as the lung biopsy (Figures 4c, d).

**Figure 5:**
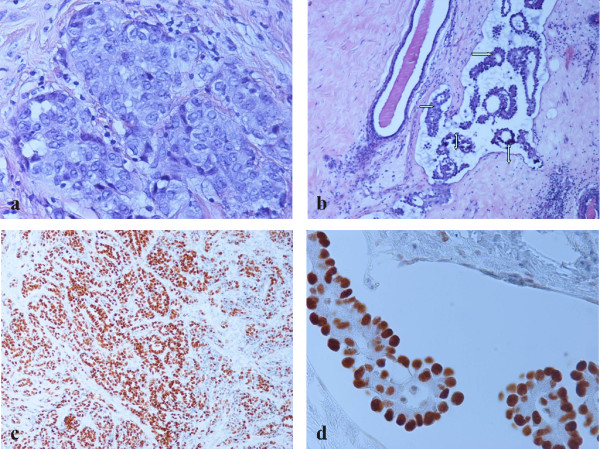
**Breast biopsy**. a) Nodular infiltration by solid (high grade) adenocarcinoma. (Hematoxylin-eosin, ×400). b) Lymphatic tumor emboli of micropapillary pattern adenocarcinoma (single arrow) and multiple psammoma bodies (double arrow). Ectatic duct is noted in the left side. (Hematoxylin-eosin, ×100). c) Immunohistochemical nuclear TTF-1 positivity of adenocarcinoma with solid and micropapillary component. (×100). d) Immunohistochemical nuclear TTF-1 positivity of micropapillary component. (×400).

Finally, the breast biopsy specimen demonstrated dense fibrohylinized stroma with atrophic terminal ductal lobular units. Within the stroma, sharply demarcated nodules of a high grade adenocarcinoma with solid and micropapillary pattern was demonstrated (Figure 5a). Lymphatic tumor emboli of micropapillary pattern adenocarcinoma with multiple psammoma bodies were also identified (Figure 5b). The surrounding breast parenchyma demonstrated mild fibrocytic changes. Finally, no evidence of in situ carcinoma or elastosis was observed. Taking into account the diagnosis of the lung and pleura biopsies our differential diagnosis included a second primary breast carcinoma and metastatic lung carcinoma. The tumor cells demonstrated immunoreactivity for TTF-1 (Figure 5c, d), SP-A and lacked expression of GCDFP-15, ER and mammaglobin (Table 1).

## Discussion

Worldwide, lung cancer is the most common cancer in terms of both incidence and mortality (1.6 million new cases per year and 1.378 million deaths) [8]. Approximately one fifth, of newly diagnosed lung adenocarcinomas, present with distant metastases. The most common sites of metastasis are brain, bone, liver, and adrenal glands, in decreasing order. However autopsy series have demonstrated that NSCLCs may spread to virtually any organ.

Breast metastases from extra-mammary malignancies are rare accounting for 0.4 to 1.3% of all breast malignancies [2-5]. Nearly 700 cases have been reported in small series and case reports [2-5,7,12,16,17].

According to the international literature the most common sources of primary tumors were haematological malignancies, malignant melanoma, lung tumors, renal cell carcinoma, ovarian tumors, thyroid carcinomas and small bowel carcinoids [3,7,17]. Williams et al in the largest published series of 169 cases with metastases to the breast from extramammary solid tumors reported that the most common histological type was malignant melanoma [7].

Review of the literature (1990-2010) includes approximately 30 NSCLCs as case reports or part of a series of secondary breast tumors [4,5,9-12,18-26]. Twelve of the above cases were classified as adenocarcinomas [5,9,12,18,20-22,24]. Additionally, 53 cases of breast metastasis from lung tumors were presented however, no detailed histological classification was provided [7,17,27-29]. The majority of breast metastases present as palpable, rapidly growing, well circumscribed, painless breast masses with predilection to the upper outer quadrant [2,7,12,16,21]. Unlike primary tumors, in the vast majority of metastases retraction of the skin or nipple is not demonstrated despite their superficial location [5,21]. However, in our patient, the lesion was poorly defined and skin redness was observed. Other authors have rarely reported similar findings [7,16,24,29].

Distinguishing a breast metastasis from a primary mammary adenocarcinoma, based on mammographic findings, may be extremely difficult because of the wide range of imaging manifestations of the metastatic lesion [4,5,17]. Thus, metastasis can mimic a primary malignancy or even a benign breast tumor [4,5,17]. The most commonly described mammographic presentation is usually single but sometimes multiple well circumscribed lesions with smooth margins [3,17,29]. Microcalcifications are very uncommon but have been reported in patients with metastatic serous ovarian papillary carcinoma [17,28,29]. In our case, mammography showed diffuse asymmetrical density and skin thickening. In cases such as ours the differential diagnosis includes inflammation, lymphoma and inflammatory breast carcinoma.

As sited in the literature, histological features that may aid in the recognition of secondary tumors are the following: The absence of in situ carcinoma strongly supports a metastatic tumor, although it may not be present in all primary invasive carcinomas. Additionally, metastatic malignancies are often sharply circumscribed from the surrounding breast tissue. Furthermore, elastosis is common in primary tumors but rare in extramammary malignancies [2,4,5,12,18]. Occasionally, metastases to the breast demonstrate features that can lead pathologists to the correct diagnosis such as presence of pigmentation and intranuclear inclusions in malignant melanomas. Nevertheless, many extramammary malignancies such as adenocarcinoma of the lung lack specific histological features.

Carcinomas with a micropapillary component have been described in many organs including the breast, urinary bladder, ovary and salivary glands [13]. Amin et al. in 2002, was the first to report lung adenocarcinomas with micropapillary component [14]. Histologically, the latter is characterized by small papillary tufts lying freely within alveolar spaces or encased within the thin walls of connective tissue. These small, cohesive nests lack fibrovascular connective tissue cores [14]. In our case all biopsies examined demonstrated an extensive micropapillary component. Although psammoma bodies have not been observed in invasive micropapillary pattern carcinoma of the urinary bladder and salivary glands they have rarely been reported in cases of lung adenocarcinoma with micropapillary morphology [13,14,30]. Multiple psammoma bodies were demonstrated in the tissue sections of our samples examined. To the best of our knowledge this is the first report of a breast metastasis from lung adenocarcinoma with micropapillary pattern diagnosed concomitantly with the primary tumor.

The distinction between metastasis from lung adenocarcinoma, particularly with extensive micropapillary pattern, and primary mammary adenocarcinoma may cause a significant diagnostic dilemma. The contribution of immunohistochemstry to the correct diagnosis is crucial.

TTF-1 is expressed in 68-80% of lung adenocarcinomas, and besides a single case published by Klingen TA et al, has never been reported to stain positive in breast adenocarcinoma [31-33]. The sensitivity of SP-A is substantially less. It is expressed in approximately 45% of lung adenocarcinomas [32,33]. A negative expression of thyroglobulin excludes the diagnosis of papillary carcinoma of the thyroid, which stains positive to both markers. ERs are expressed in 80% and GCDFP-15 in 45-53% of breast carcinomas [32,34]. As recently published, ER expression in the lung adenocarcinoma, by using the monoclonal antibodies 1D5 and 6F11 is low (7,6-14,1%) [32,35]. Additionally, 5,2-15% of lung adenocarcinomas express GCDFP-15 [34,36]. Finally, mammaglobin is expressed in 48-72,1% of mammary adenocarcinomas but stains negative in pulmonary adenocarcinomas [32,34,37]. Consequently, a panel of markers must be used as no single antibody is 100% sensitive and false negative results do occur. In our case, all the tumor specimens (lung, pleura and breast) showed positive nuclear staining for TTF-1 and cytoplasmic staining for SP-A. The neoplastic cells lacked expression of GCDFP-15, ER and mammaglobin.

Overall metastasis to the breast has been associated with poor prognosis with most patients dying within a year of diagnosis [7]. Our patient survived 6 months following the diagnosis of both the primary lung tumor and the breast metastasis.

## Conclusions

We reported a rare case of metastasis to the breast from an adenocarcinoma of the lung with extensive micropapillary component. Metastatic disease to the breast, although rare, should be considered in the differential diagnosis of a primary mammary carcinoma because the treatment and prognosis differ significantly. Furthermore, the distinction between metastasis from lung adenocarcinoma, particularly with extensive micropapillary pattern, and primary breast adenocarcinoma may cause a significant diagnostic dilemma. The contribution of immunohistochemstry to the correct diagnosis is very important.

## Consent

Written informed consent was obtained from the patient for publication of this case report and accompanying images. A copy of the written consent is available for review by the Editor-in Chief of this Journal.

## Competing interests

The authors declare that they have no competing interests.

## Authors' contributions

NM and MC designed the study, performed the cytological, histological and immunohistochemical evaluation, literature review and drafted the manuscript.

SL participated in histological diagnosis and immunohistochemical evaluation.

EE participated in cytological diagnosis.

AE and MD participated in revising the manuscript.

XT conceived the study and provided the clinical data.

All authors read and approved the final manuscript.
